# Supplementary material for: Comparative Analysis of Lymphocyte Populations in Post-COVID-19 Condition and COVID-19 Convalescent Individuals
Source: Diagnostics (Basel). 2024 Jun 18;14(12):1286. doi: 10.3390/diagnostics14121286 (PMC11202600; doi:10.3390/diagnostics14121286)
Supplement: Supplementary file 1 [file diagnostics-14-01286-s001.zip › TableS1.docx]

Supplementary Table 1: Description and specification of antibodies used for phenotyping of diagnostic panels

| **Antigen** | **Fluorochrome** | **Clone** | **Manufacturer** |
| --- | --- | --- | --- |
| *General lymphocyte subsets* | | | |
| CD45 | APC-H7 | 2D1 | Becton Dickinson |
| CD3 | V500 | UCHT1 | Becton Dickinson |
| CD4 | FITC | SK3/SK4 | Becton Dickinson |
| CD8 | PerCP-Cy5.5 | SK1 | Becton Dickinson |
| CD19 | APC | HIB19 | Becton Dickinson |
| TCRγδ | APC | B1 | Becton Dickinson |
| CD38 | PE | HIT2 | Becton Dickinson |
| CD16 | PE-Cy7 | 3G8 | Becton Dickinson |
| CD56 | PE-Cy7 | B159 | Becton Dickinson |
| *B cell subpopulations* | | | |
| CD19 | V500 | HIB19 | Becton Dickinson |
| CD20 | FITC | 2H7 | Becton Dickinson |
| CD21 | PE-Cy7 | B-ly4 | Becton Dickinson |
| CD138 | PerCP-Cy5.5 | MI15 | Becton Dickinson |
| CD27 | BV421 | M-T271 | Becton Dickinson |
| CD38 | APC | MI15 | Becton Dickinson |
| IgG | APC-Vio770 | IS11-3B2.2.3 | Miltenyi Biotech |
| IgA | APC-Vio770 | IS11-8E10 | Miltenyi Biotech |
| IgA | PE | IS11-8E10 | Miltenyi Biotech |
| IgM | PE | PJ2-22H3 | Miltenyi Biotech |
