# Supplementary material for: Comparative Analysis of Lymphocyte Populations in Post-COVID-19 Condition and COVID-19 Convalescent Individuals
Source: Diagnostics (Basel). 2024 Jun 18;14(12):1286. doi: 10.3390/diagnostics14121286 (PMC11202600; doi:10.3390/diagnostics14121286)
Supplement: Supplementary file 1 [file diagnostics-14-01286-s001.zip › TableS2.docx]

Supplementary Table 2: Comparison of indicative laboratory data form COVID-19 patients grouped by first contact

|  | COVID-19  All | | COVID-19  Group 1 (G1) | | COVID-19  Group 2 (G2) | | COVID-19  Group 3 (G3) | |
| --- | --- | --- | --- | --- | --- | --- | --- | --- |
|  | Acute phase | Follow-up | Acute phase | Follow-up | Acute phase | Follow-up | Acute phase | Follow-up |
| N | 73 | | 21 | | 38 | | 14 | |
| Lymphocytes in Gpt/l  (IQR) | **0.96**  **(0.76-1.27)** | 1.75  (1.45-2.31) | **0.86**  **(0.76-1.28)** | 1.91  (1.61-2.47) | **1,02**  **(0.83-1,1)** | 1.71  (1.44-2.39) | **0,76**  **(0.65-1,04)** | 1.76  (1.35-2.38) |
| Neutrophils in Gpt/l  (IQR ) | 4.04  (3.18-5.80) | 4.16  (3.48-5.40) | **3.84**  **(3.39-5.44)** | 4.68  (3.78-5.94) | 4.4  (3.15-4.60) | 4.14  (3.50-4.45) | 4.4  (3.15-4.60) | 3.96  (3.13-4.43) |
| Neutrophil lymphocyte Ratio  (IQR) | **4.17**  **(3.22-6.49)** | 2.14  (1.68-3.28) | **4.61**  **(3.39-5.83)** | 1.89  (1.67-3.21) | **3.89**  **(3.21-6.22)** | 2.19  (1.78-3.24) | **5.71**  **(3.84-6.84)** | 2.11  (1.58-3.25) |
| C reactive Protein in mg/l  (IQR) | **40.7**  **(20.1-69.8)** | 2.3  (1.1-3.7) | **36.1**  **(17.3-67.6)** | 1.6  (0.8-3.1) | **50.2**  **(25.2-69.4)** | 2.3  (1.1-4.7) | **32.9**  **(21.5-87.7)** | 2.9  (2.1-5.9) |

Values are given as Median with interquartil range (IQR). Significant difference (p<0.05) applying the non-parametric Mann-Whitney-Wilcoxon test are highlighted in bold.
