# Supplementary material for: Comparative Analysis of Lymphocyte Populations in Post-COVID-19 Condition and COVID-19 Convalescent Individuals
Source: Diagnostics (Basel). 2024 Jun 18;14(12):1286. doi: 10.3390/diagnostics14121286 (PMC11202600; doi:10.3390/diagnostics14121286)
Supplement: Supplementary file 1 [file diagnostics-14-01286-s001.zip › TableS3.docx]

Supplementary Table 3: Comparison of indicative laboratory data form COVID-19 patients grouped by final outcome

|  | COVID-19  All | | COVID-19  NPCC | | COVID-19  PCC | |
| --- | --- | --- | --- | --- | --- | --- |
|  | Acute phase | Follow-up | Acute phase | Follow-up | Acute phase | Follow-up |
| N | 73 | | 26 | | 47 | |
| Lymphocytes in Gpt/l  (IQR) | **0.96**  **(0.76-1.27)** | 1.75  (1.45-2.31) | **1.04**  **(0,82-1,51)** | 1.92  (1.58-2.43) | **0.94**  **(0.715-1.13)** | 1.71  (1.41-2.26) |
| Neutrophils in Gpt/l  (IQR ) | 4.04  (3.18-5.80) | 4.16  (3.48-5.40) | 4.87  (3.64-6.03) | 4.11  (3.49-4.78) | 3.83  (2.92-5.49) | 4.17  (3.45-5.41) |
| Neutrophil lymphocyte Ratio  (IQR) | **4.17**  **(3.22-6.49)** | 2.14  (1.68-3.28) | **4.30**  **(2.42-6-78)** | 2.16  (1.73-3.23) | **4.12**  **(3.31-5.75)** | 2.14  (1.68-3.37) |
| C reactive Protein in mg/l  (IQR) | **40.7**  **(20.1-69.8)** | 2.3  (1.1-3.7) | **36.05**  **(16.2-68.9)** | 1.55  (1.03-3.03) | **43.3**  **(24.5-70-2)** | 2.6  (1.2-4.6) |

Values are given as Median with interquartil range (IQR). Significant difference (p<0.05) applying the non-parametric Mann-Whitney-Wilcoxon test are highlighted in bold.
