# Supplementary figures and images for: Comparative Analysis of Lymphocyte Populations in Post-COVID-19 Condition and COVID-19 Convalescent Individuals
Source: Diagnostics (Basel). 2024 Jun 18;14(12):1286. doi: 10.3390/diagnostics14121286 (PMC11202600; doi:10.3390/diagnostics14121286)

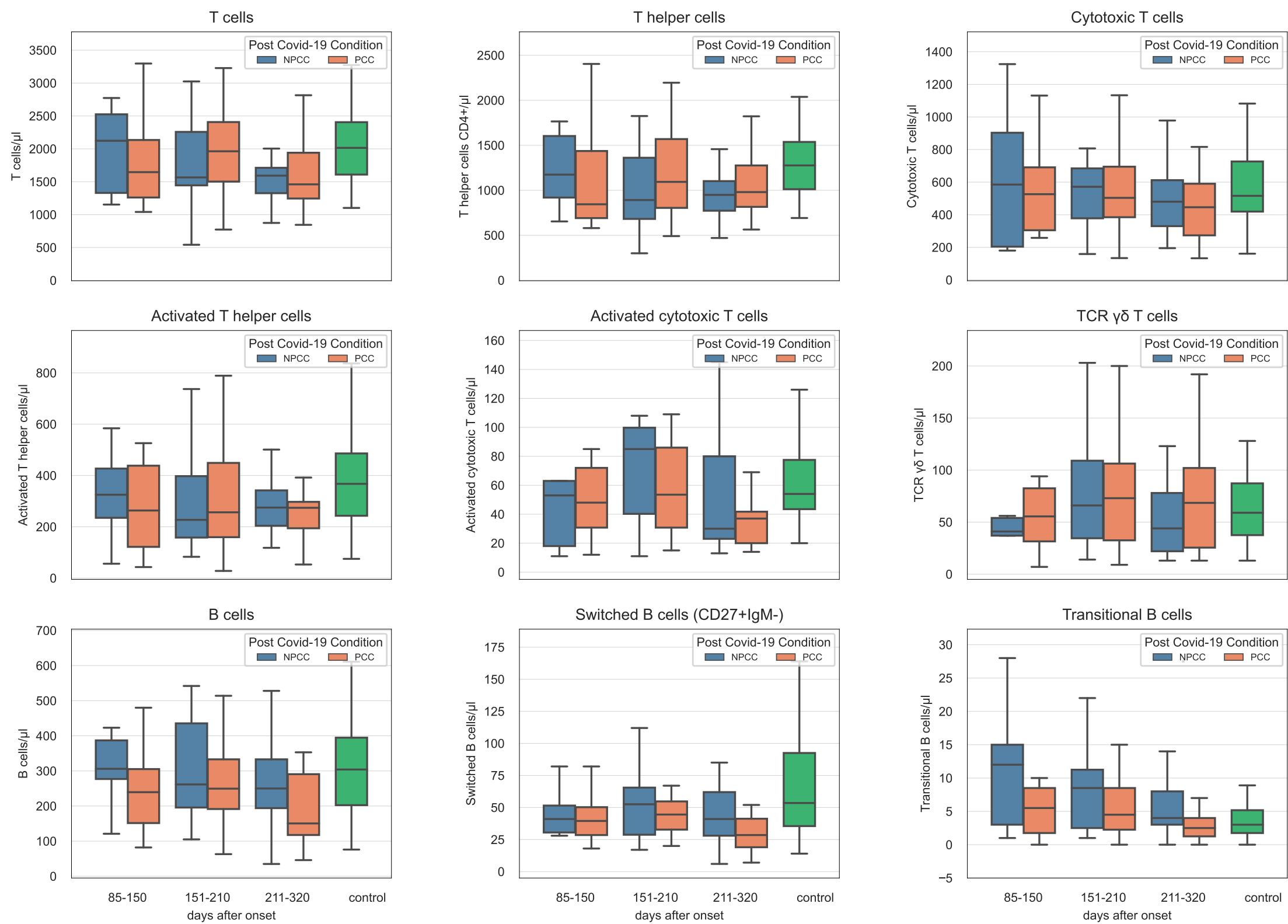

Supplement: Supplementary file 1 [file diagnostics-14-01286-s001.zip › FigureS1.pdf]

CD21low B cells

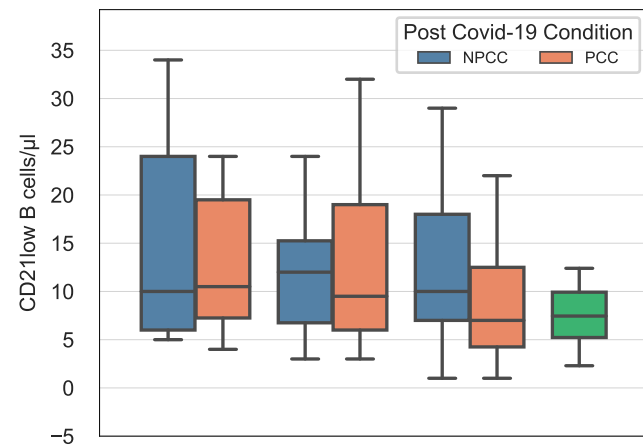

IgM+CD27- B cells

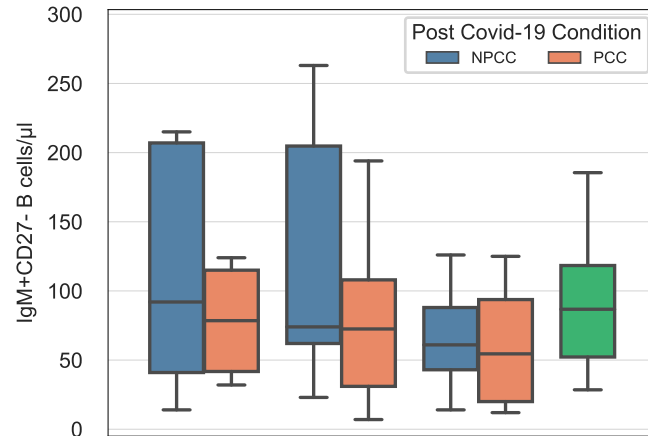

IgA+CD27+ B cells

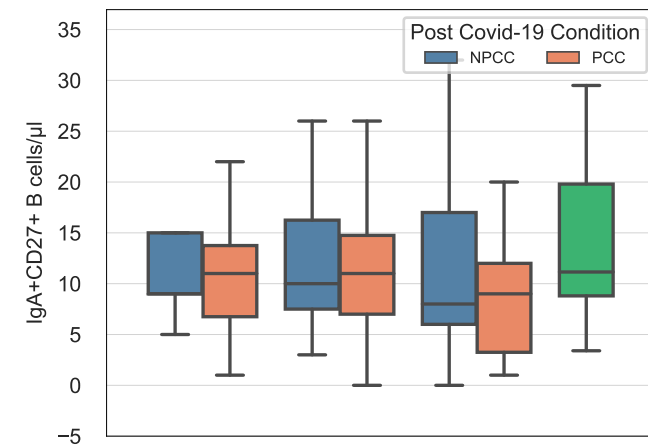

IgG+CD27+ B cells

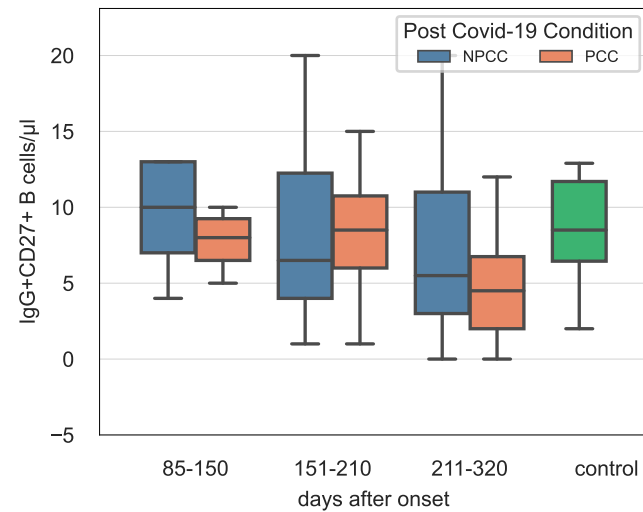

NK cells

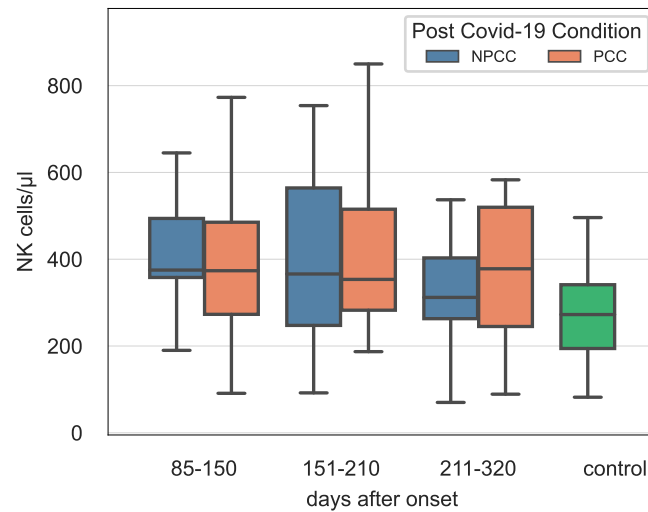

NKT cells

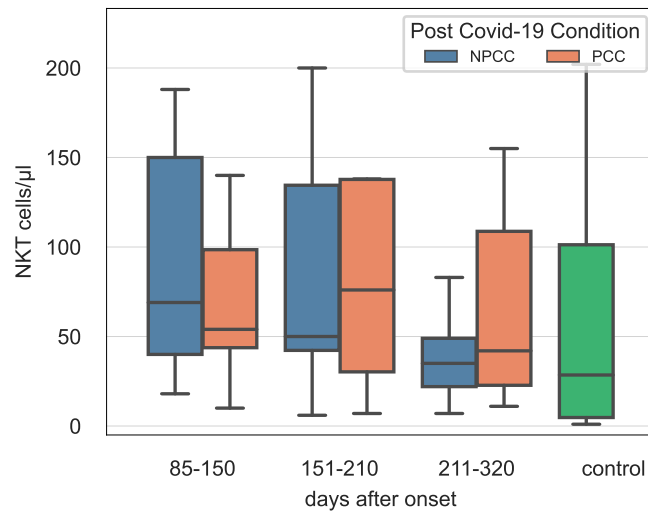

Supplement: Supplementary file 1 [file diagnostics-14-01286-s001.zip › FigureS2.pdf]
